# Comparative approaches to gentrification: Lessons from the rural

**DOI:** 10.1177/2043820617752009

**Published:** 2018-02-26

**Authors:** Martin Phillips, Darren P Smith

**Affiliations:** University of Leicester, UK; Loughborough University, UK

**Keywords:** comparative urbanism, France, rural gentrification, United Kingdom, United States, urban gentrification

## Abstract

The epistemologies and politics of comparative research are prominently debated within urban studies, with ‘comparative urbanism’ emerging as a contemporary lexicon of urban studies. The study of urban gentrification has, after some delay, come to engage with these debates, which can be seen to pose a major challenge to the very concept of gentrification. To date, similar debates or developments have not unfolded within the study of rural gentrification. This article seeks to address some of the challenges posed to gentrification studies through an examination of strategies of comparison and how they might be employed within a comparative study of rural gentrification. Drawing on Tilly (*Big structures Large Processes Huge Comparisons*. New York: Russell Sage), examples of four ‘strategies of comparison’ are identified within studies of urban and rural gentrification, before the paper explores how ‘geographies of the concept’ and ‘geographies of the phenomenon’ of rural gentrification in the United Kingdom, United States and France may be investigated using Latour’s (*Pandora’s Hope*. London: Harvard University Press) notion of ‘circulatory sociologies of translation’. The aim of our comparative discussion is to open up dialogues on the challenges of comparative studies that employ conceptions of gentrification and also to promote reflections of the metrocentricity of recent discussions of comparative research.

## Introduction

There is a growing interest in comparative research, particularly in urban studies where comparative urbanism is a vibrant subject of discussion (McFarlane and Robinson, 2012; Robinson and Roy, 2016; Ward, 2010), albeit one that has not hitherto featured in *Dialogues in Human Geography*. Here we rectify this omission by explicating the application of these debates to one research area where comparative research is prominent, namely the study of gentrification.

As Bernt (2016: 1) observed, the arrival of comparative urbanism into gentrification scholarship raises challenges whose relevance constitutes ‘a turning point not only for gentrification research, but also for the way we develop established concepts into a more global body of knowledge’. Bernt highlights how the rise of comparative research has led to an expansion in the geographical focus of gentrification studies, with attention paid to spatial variabilities in the concept, form and extent of gentrification. As Lees (2012: 157–158) comments, this interest preceded the emergence of the notion of comparative urbanism, with gentrification researchers having a long-standing interest in how ‘theories of gentrification have travelled and how the process itself has travelled’. She adds that different forms of gentrification emerge ‘in different places at different and indeed the same times’ and that meanings associated with gentrification in one place may not translate easily, if at all, to other locations. Consequently, she argues, researchers need to ‘critically debate the international significance of the term “gentrification”’ and ‘consider how comparison might take place’ (Lees, 2012: 158). As such, gentrification research might be commensurable and reinvigorated by interest in comparative research. Yet, as Bernt (2016: 1) observes, the rise of comparative research has led to calls for abandonment of the gentrification concept, with Ghertner (2015: 522) wondering whether it is now ‘time to lay the concept to bed’. Bernt (2016: 1), while drawing back from such arguments, sees value in some of Ghertner’s claims and observes that the impact of comparative research on gentrification is ‘an increasingly open question’.

We address this question via consideration of the potential and value of comparative research on rural gentrification. While identified as a somewhat ‘neglected other’ to the study of urban gentrification (Phillips, 2004), recent decades have seen increasing reference to rural gentrification, especially in the United Kingdom (e.g. Phillips, 2002; Smith, 2002a; Stockdale, 2010) and North America (e.g. Darling, 2005; Hines, 2012; Nelson and Nelson, 2010), but also in other countries (e.g. Hjort, 2009; Qian et al., 2013; Solana-Solana, 2010). There are, however, many countries where there has been little use of the concept, and even in places where it has been employed, rural gentrification remains a minor motif within rural geography and a peripheral constituent of wider gentrification debates. Theorizing from positions of marginality has been a major point of argument within elaborations of comparative urbanism (e.g. McFarlane, 2010; Roy, 2009, 2016), and we want to stimulate consideration of the extent to which framings other than the urban might contribute to elaborating comparative studies of gentrification. More specifically, we explore how a comparative study of rural gentrification in France, United Kingdom and United States could be developed to engage with the challenges identified by Bernt (2016).

To develop its arguments, the article begins by considering strategies of comparison as outlined within comparative urbanism and explores how these have been performed within urban gentrification studies. Hitherto, discussions of comparative approaches within these studies have been narrow in focus, particularly when set alongside the literature on strategies, practices and politics of comparison associated with comparative urbanism. Drawing on Tilly (1984) and Robinson (2015), we suggest that practices of comparison enacted in gentrification studies are more diverse than are represented in existing literatures. From this starting point, the article argues that the strategies of comparison identifiable within urban gentrification studies are present within rural studies, albeit with differences in extent and focus. The article then focuses on a comparative study of rural gentrification in France, United Kingdom and United States, drawing on the concept of ‘sociologies of translation’, outlined by Latour (1999), to explore both the ‘geographies of the concept’ and ‘geographies of the phenomenon’ of rural gentrification (Clark, 2005). The article concludes by considering relationships between these two geographies of rural gentrification and strategies of comparison.

## Comparative urbanism and urban gentrification

Comparative urbanism highlights the prevalence and complexity of comparison. Ward (2010: 473), for example, argues that ‘comparison is practically omnipresent in much empirical social science research’, while McFarlane (2010: 725) asserts that theoretical abstractions inevitably, albeit often implicitly, make comparative assertions, because ‘claims and arguments are always set against other kinds of…possibilities or imaginaries’. Practices such as literature citation, for example, set up comparisons with existing bodies of knowledge. McFarlane claims that comparative practices should be explicitly discussed, with consideration paid to both epistemological methodologies and the politics of comparison. The former involves consideration of the practicalities of comparison, such as language, resources, the delimitation of scope and focus, methods of comparison and the role and construction of comparative typologies.

In relation to this last feature, Lagendijk et al. (2014) argue that comparative studies of gentrification often focus on establishing a metric to actualize interpretations and practices across spatial contexts. Examples include studies by Ley (1986; 1988; 2003) and Wyly and Hammel (1998; 2004), which variously illustrate difficulties in constructing comparative metrics, including ‘readily available secondary data’ (Wyly and Hammel, 1998: 305) failing to map onto conceptual arguments and/or be available across localities being compared (Ley, 1996).

Metric-based analysis could be characterized as fitting within McFarlane and Robinson’s (2012: 767) description of ‘quasi-scientific’ research focused on the identification of a narrow range of comparative traits, an approach they claim is ‘inappropriate’ given the ‘multi-dimensional, contextual, interconnected, and endogenous nature of urban processes’. In the context of gentrification research, Lees et al. (2015b: 9) similarly argue that structured comparative approaches ‘flatten cases’ through focusing on ‘a limited number of factors or categories’. They make no use of metric-based analysis, but rather propose practices of transnational ‘collegiate knowledge production’ (Lees et al., 2015b: 13; see also López-Morales et al., 2016). However, Lagendijk et al. (2014: 362) utilize assemblage theory to propose that, rather than either foster the articulation of generalized metrics or reject them as being ‘untrue to reality’, comparative studies of gentrification might recognize their presence within the ‘worlds of gentrification’ and study their ‘actualisation and counter-actualisation’ within a range of localities. This is a productive position, although it implies that comparative studies would only examine spaces where metrics were present, which might severely limit the scope of such studies.

A range of positions on the value of metrics and typologies to comparative studies are being advanced within gentrification studies, although, as yet, there remains little sustained discussion of their epistemological significance or the practices required for alternative strategies of comparison. There is a significant difference here between discussions of comparative studies of gentrification and the literature on comparative urbanism which contains much greater epistemological reflection, with Tilly’s (1984) identification of ‘individualising’, ‘universalising’, ‘encompassing’ and ‘variation-finding’ strategies (Table 1) being widely cited (e.g. Brenner, 2001; Robinson, 2011). We demonstrate that these have applicability to gentrification studies and, hence, can advance the development of comparative studies of gentrification, although as the article develops we layer in other understandings of comparison, derived from comparative urbanism and studies of gentrification.

**Table 1.** Comparative approaches in gentrification research.

Share of instances	Multiplicity of forms Single ↔ Multiple	
One ↕ All	*Individualizing* Carpenter and Lees (1995); Lees (1994); Maloutas (2012)	*Encompassing* Smith (1982; 1996; 2002)
	*Universalizing* Ley (1996); Smith (1996); Hackworth and Smith (2001); Hackworth (2007)	*Variation-finding* Van’s Gent (2013); Hochstenbach (2015).

### Individualizing and variation-finding gentrification studies

Ward (2010) suggests that individualizing and variation-finding strategies characterize much of comparative urban studies. The focus in the former is on comparing instances of a phenomenon to identify the particularities of each case. Gentrification examples include the comparisons of Carpenter and Lees (1995: 286) focused on a ‘questioning of generalizations about the gentrification process and an emphasis on international differences’, Musterd and van Weesep’s (1991) examination of whether gentrification in Europe was an instance of a generalized process or involved specifically European dynamics, and Butler and Robson’s (2003) study of neighbourhoods in London that emphasized the different compositions of gentrifiers in each locality.

A recent, and epistemologically focused, example is Maloutas’ (2012) criticism of the application of the concept of gentrification across contexts. He claims that this, first, leads to a decontextualization of the concept, which becomes increasingly abstract in order to be applicable across cases. An illustration is Clark’s (2005: 258) creation, by ‘realist abstraction’, of ‘an elastic yet targeted definition’ of gentrification, an argument employed in developing the notion of ‘generic gentrification’ (Hedin et al., 2012). Maloutas (2012: 38–39) asserts, however, that abstract conceptions of gentrification produce a neglect of ‘causal mechanisms and processes’, in favour of a superficial focus on ‘similarities in outcomes across contexts’.

Second, Maloutas argues that while gentrification scholars have sought to decontextualize the concept, it remains marked by the context of creation. Specifically, he contends that the concept was developed in, and was of considerable significance in understanding changes within, cities such as London and New York. Attempts to make the concept travel to other time-spaces are, he claims, flawed because conditions in these contexts are different. Third, he argues that attempts to make gentrification travel are ideological, acting to project ‘neoliberal framings’ across contexts.

Maloutas is an exponent of individualizing comparison, viewing concepts as inextricably linked to contexts. Such arguments when advanced within comparative urbanism have been subject to criticism, with Peck (2015: 179) commenting that such work can be particularist rather than comparative, promoting ‘hermetically sealed’ modes and sites of analysis. With respect to gentrification, Lees et al. (2015b: 7) state that Maloutas creates ‘fossilisation not contextualisation’, reifying the ‘contextual epiphenomena’ of gentrification, such as how it ‘looked, smelled or tasted in some specific (North American and West European) contexts at very specific times’, to create a simplified and static conception of gentrification that cannot be reasonably applied beyond its initial context. They add that while there are lessons to be learnt from comparative urbanism, ‘we should not throw the baby out with the bathwater’ (Lees et al., 2015b: 9) and seek to ‘stand aside’ from a ‘flat ontology’ dedicated to the appreciation of difference in favour of an ontology focused on ‘social injustices and power relations’. It is further asserted, ‘that a large number of well analysed cases help extract global regularities of the causes of gentrification’ (Lees et al., 2015b: 6).

While few gentrification researchers hitherto appear willing to fully embrace Maloutas’ individualizing perspective, many studies implicitly employ it by drawing comparisons to pre-existing studies to emphasize the particularities of their study. Instances of variation-finding comparisons, which are identified by Tilly (1984) as strategies that seek to identify causes of variation across cases, include van Gent’s (2013) ‘contextual institutional approach’, which, although focused on Amsterdam, explains variations from other studies of ‘third-wave gentrification’ via institutional practices (see also Hochstenbach et al., 2015).

### Universalizing and encompassing comparisons

While individualizing and variation-finding comparisons can be identified in gentrification studies, universalizing and encompassing perspectives have a stronger presence. Universalizing comparisons focus on establishing that instances share common, and generally independently constituted, properties, with change within them viewed as largely driven by dynamics internal to these cases. The approach generally enacts ‘an incipient monism’ (Leitner and Shepherd, 2016: 231) in that certain features are seen to be significant to all the identified cases, and universalizing comparisons also often adopt ‘developmentalist perspectives’, with differences between cases viewed as reflections of differential positions within a common path.

Examples of universalizing perspectives can be identified within gentrification studies. Early decades of gentrification studies, for example, involved ‘legislative’ debates (Phillips, 2010) concerning the applicability of various monist conceptions of gentrification to a widening number of cases. For authors such as Lambert and Boddy (2002), the spatial extension of locations identified as undergoing gentrification stretched the term to encompass so much difference that, as per Moulatas, it lost any specific meaning. For others, commonalities could be discerned within such differences. Reference has already been made to Clark’s (2005: 260–261) adoption of realist abstraction, and he sought to use this to identify both generic ‘underlying necessary relations and causal forces’ associated with gentrification and features which, while crucially significant in understanding the formation and impact of gentrification in particular localities, were contingent in character. Recent years have seen a series of applications of these arguments to comparative studies of gentrification (Betancur, 2014; Lees et al., 2016; López-Morales, 2015; Shin et al., 2016). A different, but related, perspective was work, such as Smith (2002b) and Lees et al. (2008), suggesting that the character of gentrification was itself changing, such that early definitions were now inappropriate to identify the presence, processes and varied forms of contemporary gentrification. Strands of continuity, such as class transformation, displacement and capital flows into built environments, were, however, also identified.

In both sets of work, the universalism of identifying continuities and/or abstract commonalities was tempered, to a degree, by recognition that gentrification could take a range of different forms. This was evident in ‘stage-theories’ of gentrification (Clay, 1979; Gale, 1979; Hackworth, 2007; Hackworth and Smith, 2001). As discussed in Phillips (2005), these interpretations have been criticized for employing developmentalist logics, whereby gentrification is framed as a singular process impacting locations which move, or in some cases fail to move, through a predetermined series of stages, although attention has been drawn to differences in trajectories of change, to instances of non-development and to the multiplicity of gentrification forms present in a location at particular points in time (Ley and Dobson, 2008; Pattaroni et al., 2012; Van Criekingen and Decroly, 2003).

Universalizing comparisons were also enacted in discussions of ‘gentrification generalised’, which often portrayed gentrification as a singular process ‘cascading’ both ‘laterally’ across national borders and ‘vertically’ down ‘the urban hierarchy’, until it reached ‘even small market towns’ (Smith, 2002b: 439) or ‘unfurled to include rural settlements’ (Atkinson and Bridge, 2005: 16). Such views encouraged the adoption of an implicit, ‘imitative urbanism’, whereby processes of urban gentrification are seen to have ‘travelled to and been copied in the Global South’ (Lees, 2012: 156). Such perspectives are viewed as ‘western-centric’ by comparative urbanists influenced by post-colonialism (e.g. Robinson, 2004; 2011), as well as by gentrification researchers such as Maloutas (2012), Lees (2012) and Lees et al. (2015a, b), who highlight how such interpretations may act as ‘deforming lenses’ (Maloutas, 2012: 43), projecting occidental concerns and assumptions at the expense of recognizing specificities and differences. However, it can also be argued that these conceptions are overly urban-centric in their focus, viewing gentrification as originating in and diffusing from a selected number of metropolitan sites to other urban and, eventually, rural sites. This imagery neglects the identification of sites of rural gentrification soon after coinage of the term gentrification by Glass (see Phillips, 1993). Just as post-colonialists have highlighted how occidental concerns may be projected over cities of the South, researchers often position the urban as ‘a privileged lens through which to interpret, to map and, indeed, to attempt to influence contemporary social, economic, political and environmental trends’ (Brenner and Schmid, 2015: 155).

Universalizing comparisons do not have to be coupled with diffusionist perspectives. Brenner et al. (2010: 202), for example, identify the possibility of ‘accumulation of contextually specific projects’, and Peck (2015: 171) argues for recognition of ‘common, cross-contextual patterns and processes’, while Robinson (2015) calls for examination of repetition as singular assemblings. In this perspective, repeated appearance is not seen as diffusion of a common process but as a series of singular outcomes of processes, practices and relations in operation within multiple localities.

Such arguments resonate with urban gentrification scholarship. Lees et al. (2015a: 442), for example, argue for recognition of the ‘transnational mobility of gentrification’ and ‘its endogenous emergence’ in a range of locations, such that gentrification may be viewed as multiple and multi-centric, although there are still said to be ‘necessary conditions’ (Lees et al., 2015b: 8) that need to be present before gentrification can be said to exist. A similar, and in our view more productive, way of framing such arguments is to suggest that universalizing comparisons be viewed as ‘genetic comparisons’ (Robinson, 2015), identifying singularly constituted transformations in locations across which there are some recurrent features viewed as constitutive of gentrification, but in each case, these will have been produced within that locality. These recurrent features might be viewed as the abstract ‘generic’ dimensions of gentrification outlined by Clark (2005), although within a genetic approach these elements would be viewed as contingently created as the other elements of each case, rather than identified as established through some form of necessary relationship. As such, the genesis of the generic dimensions requires explanation in each instance rather than being viewed as foundationally determinant. Furthermore, while each case may involve, or be stimulated by, movement of resources and agents into that locality from beyond, it is likely that there will be at least some spatially and/or temporally specific elements. Such an approach would counter the monism and developmentalism that has been the focus of criticism.

The final form of comparison identified by Tilly is ‘encompassing’. Here, the aim is to situate instances of a phenomenon in relationship to each other, in such a way that their form can be seen to be in large part determined by such relationships. Such understandings can be clearly identified within gentrification studies. Examples include Smith’s (1982, 1996) conceptualization of gentrification as a facet of uneven development and the globalization of gentrification (Smith, 2002b). In this latter work, Smith argues that gentrification has become global as various forms of capital sought to restructure new localities in their search for continuing profitability, with the vertical and lateral dispersal of gentrification discussed earlier, being seen to stem from an ‘influx of new capital’ into gentrification projects and disinvestment and reinvestment of existing capitals from one area to another. Similarly, Atkinson and Bridge (2005) suggest that the ‘unfurling’ of gentrification in an increasing range of spaces, including rural areas, is the result of flows of finance, people, information and ideas from one gentrified area into another (see also Lees, 2006; 2012). More recently, Lees et al. (2016: 13) have identified their examination of ‘planetary gentrification’ as ‘a relational comparative approach’ involving investigation of how instances of gentrification are ‘increasingly interconnected’. Emphasizing connections rather than similarities between cases of gentrification, these studies can be viewed as advocating encompassing rather than universalizing comparisons, although failing themselves to recognize these differences. Attention also needs to be paid to the status of these connections, with Robinson (2011) promoting use of the term ‘incorporating comparisons’ to recognize the significance of what she would later describe as the genetic elements of relational connections, that is recognizing their genesis as well as consequences.

### Politics of comparison

In addition to fostering discussion of epistemology, comparative urbanism also highlights the politics of comparison. McFarlane (2010: 726), for example, argues that comparison is a political mode of thought because it can be employed ‘as a means of situating and contesting existing claims…expanding the range of debate, and informing new perspectives’. Comparative urbanism has been particularly associated with postcolonial perspectives (e.g. Robinson, 2004, 2011), it being claimed that comparison fosters the creation of ‘readings of theory and the city’ (McFarlane, 2010: 735) less marked by the cities and urban theorists of the North. Lees (2012: 155–159) draws heavily upon this argument, claiming that ‘gentrification researchers need to adopt a postcolonial approach’. She suggests that work is needed on the mobilities and consequences of ideas of gentrification and on forms and practices of contemporary gentrification, with a key focus being postcolonial informed studies of urbanism in the Global South, although adds that ‘there remain important comparative studies to be made not just between the Global North and Global South’ (Lees 2012: 157–158).

The remainder of this article explores the potential and value of comparative studies of rural gentrification, which, as mentioned earlier, have been identified as a neglected other to the study of urban gentrification (Phillips, 2004). Indeed, while postcolonial comparative urbanists have challenged ‘metrocentricity’, where this is understood as involving a concentration of research on metropolitan centres in the Global North (Bunnell and Maringanti, 2010), the term might also be viewed in urban and rural registers as well. Thomas et al. (2011) have argued that ‘a defining element of social science education for a former inhabitant of Rural America is an overwhelming sense that you are ignored by your discipline’, a comment that echoes Lobao’s (1996: 3) commentary, although she argued that the study of rural space was not only often marginalized as the ‘non-metropolitan’ but that such a positioning could be a location of ‘creative marginality’ from which to transform the mainstream.

The following section considers how comparative strategies outlined with respect to urban gentrification relate to studies of rural gentrification. We then explore how these strategies can be deployed in comparative studies of rural gentrification in France, United Kingdom and United States, drawing on Latour’s (1999) concept of ‘circulatory sociologies of translation’ to illuminate the geographies of gentrification and geographies of ‘articulating gentrification’.

## Comparative studies of rural gentrification

Nelson et al. (2010) argue that rural gentrification studies are marked by localized case studies, with little examination of the distribution or processes of gentrification beyond these locations. This does not mean, however, that comparisons have been absent from rural gentrification studies. Reference has been made to the arguments of McFarlane (2010) that even localized studies make comparative claims, even if individualizing in character. Many rural gentrification studies include cautionary remarks concerning the transfer of ideas of gentrification from urban to rural contexts. Smith and Phillips (2001: 457), for example, coined the term ‘rural greentrification’, both to stress the ‘demand for, and perception of, ‘green’ residential space from in-migrant’ gentrifier households and to suggest that this feature ‘stands in contrast to the ‘urban’ qualities which attract in-migrant counterparts in urban locations’. Smith (2011: 603) later argues that studies reveal ‘more and more incommensurabilities between urban and rural gentrification’, while Guimond and Simiard (2010) assert that while rural researchers have drawn inspiration from urban gentrification studies, ‘important nuances must be taken into consideration when applying urban theories of gentrification to a rural context’. The significance of contextual differences has been highlighted not simply with respect to urbanity and rurality, but within the rural: Darling (2005: 1015) argues that rural areas may be ‘sufficiently differentiated to render the idea of an overarching, homogeneous “rural gentrification” suspect’, indicating a need for ‘a more refined and specific set of labels to indicate a variety of landscape-specific gentrification models’. Consideration might also be paid to the scale of landscape forms and how these connect to particular theorizations of gentrification.

**Figure 1.**
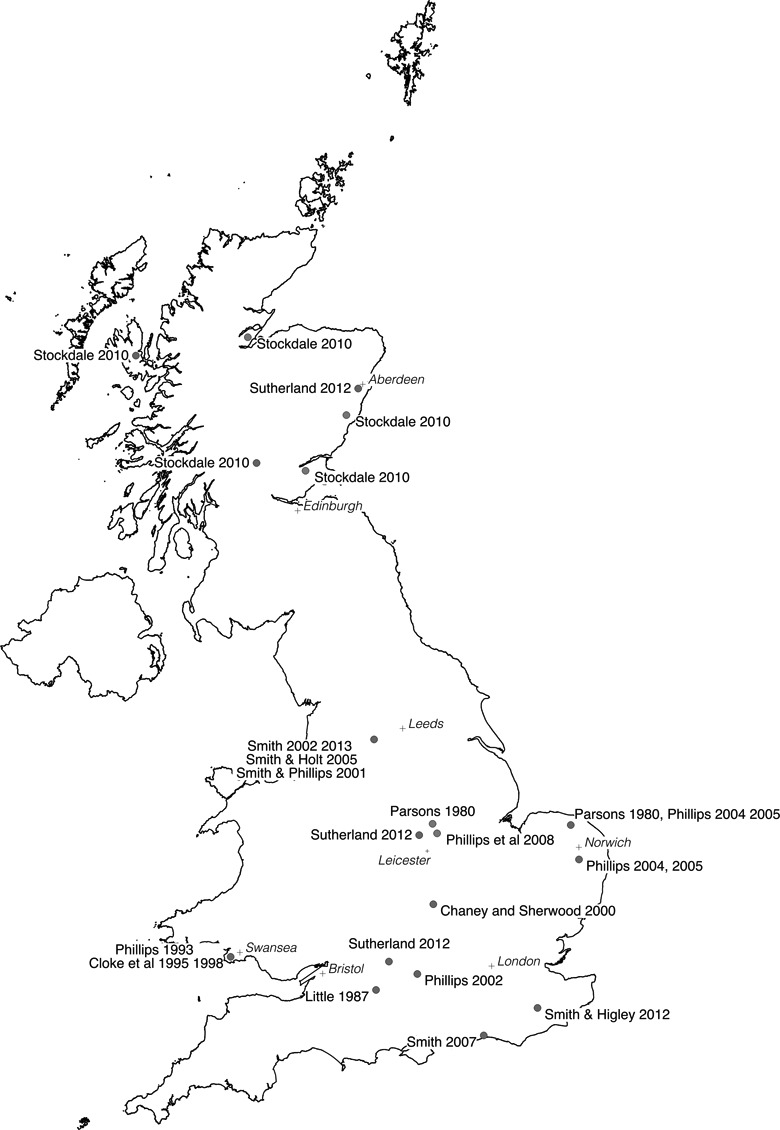
Studies of rural gentrification in the United Kingdom.

**Figure 2.**
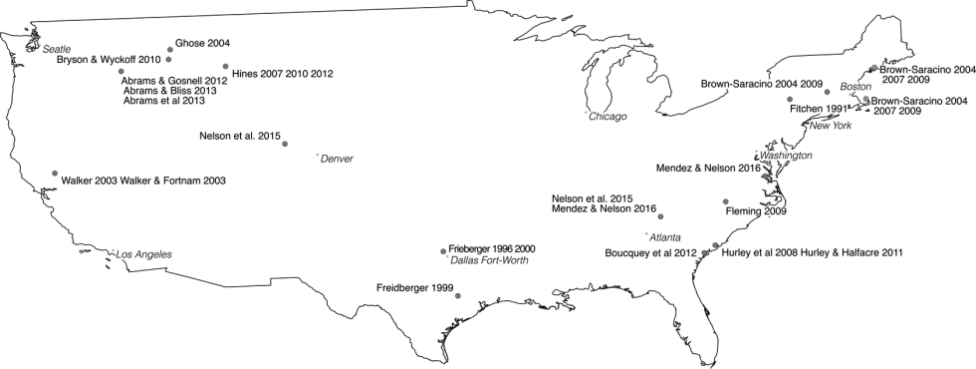
Studies of rural gentrification in the United States.

Contextual factors are significant to variation-finding as well as individualizing comparisons. The limited number of rural gentrification studies limits the scope for variation-finding comparisons, although it is possible to identify practices and processes that could cause variations in the gentrification of rural localities. As in urban contexts, governmental regulations and development controls are identified as agencies within the gentrification of rural localities (Gkartzios and Scott, 2012; Hudalah et al., 2016; Shucksmith, 2011) and clearly can be enacted differentially. Likewise, the nature and extent of rural space might condition the presence and/or form of rural gentrification (Darling, 2005; Phillips, 2005; Smith and Phillips, 2001), given differences are evident in the character of areas identified as experiencing rural gentrification: UK studies often focus on localities with extensive commuting, while North American studies tend to be in areas seen to be beyond extensive metropolitan influences (Figures 1 and 2).

Nelson et al.’s (2010) and Nelson and Nelson’s (2010) examinations of rural gentrification across the United States provide arguments for the adoption of both universalizing and encompassing comparisons. In connection to the former, Nelson et al. (2010) review existing research on rural gentrification in the United Kingdom, Spain and Australia, in order to identify mappable indicators of gentrification in non-metropolitan areas. This strategy assumes that processes of gentrification have high uniformity across rural contexts, an approach also adopted in Nelson and Nelson (2010). However, this study also enacts an encompassing focus, identifying relational reasons for moving beyond localized case studies. Globalization is viewed as a major driver of rural gentrification because key constituents of urban to rural movements are middle and upper-middle classes who have benefited from globalized capital accumulation and rising land and property values. Nelson and Nelson argue that this positioning in global capital enables these classes to acquire the assets to locate in high-amenity destinations, with gentrification in these remote rural locations being consequential to relationships with, and within, a globalized economy. Nelson et al. (2015) repeat this argument, asserting that rural gentrification in amenity areas of the United States reflects a spatial fix of surplus capital accumulated in high wage urban-based careers in the globalized service sector.

Similar arguments, albeit focused on UK rural restructuring through the settlement of a commuting ‘service class’, were advanced by Cloke and Thrift (1987), who claimed this movement was driven by changes in the international division of labour. Cloke et al. (1991) also drew attention to how movements of this class could connect into flows of exogenous ‘footloose’ capital, while Phillips (2002; 2005) stressed flows of capital from agriculture and service provision into the gentrification of properties, as well as flows of labour power, ideas and people. Nelson and Nelson (2010) and Nelson et al. (2015) identify further global connections, with the gentrification of remote amenity locations stimulating movement of low-income Latino populations to, or more often in proximity to, these localities. Parallels with studies of service class migration to accessible UK rural areas can be seen, with Cloke and Thrift (1987: 328) arguing that rural service class growth entails ‘growth of members of other classes and class fractions needed to service the service class’.

Rural gentrification studies, like their urban counterparts, enact all four strategies of comparison identified by Tilly (1984: 145), an unsurprising finding given he argues that each strategy of comparison ‘have their uses’. Both Ward (2010) and Robinson (2011) have asserted that individualizing comparisons are among the most widespread form of comparison conducted in urban studies, and this appears to be the case also in rural gentrification studies, in part because of the predominance of localized case studies. Adoption of such a strategy provides an implicit critique of universalizing perspectives, although such viewpoints are evident in rural gentrification studies, as are encompassing comparisons. Variation-finding perspectives on rural gentrification are least developed, due in part to the lack of studies from which this approach could draw. All the identified strategies of comparison, and reflections on the value of comparative studies of rural gentrification, could clearly benefit from explicit examples of comparative research. The final section of this article explores how such studies could be developed by considering how a comparative study of rural gentrification could be pursued in France, United Kingdom and United States. In undertaking this, it will draw upon the concept of sociologies of translation as outlined by Latour (1999).

## Comparing rural gentrification in France, United Kingdom and United States

The United Kingdom and United States have more extensive literatures on rural gentrification, stemming back at least to the late 1970s/early 1980s (Cloke, 1979; Lapping et al., 1983; Parsons, 1980). In France, by contrast, gentrification appears largely absent ‘from the vocabulary of French social science’ (Fijalkow and Preteceille, 2006: 6), although from the late 1990s, there was some engagement by urban researchers (Authier, 1998; Bidou, 2003; Lacour and Puissant, 2007; Préteceille, 2007) and from the 2000s in rural studies (Cognard, 2006; Perrenoud, 2008; Puissant, 2002; Richard et al., 2014).

A comparative study of rural gentrification in France, United Kingdom and United States provides an opportunity to explore reasons for, and consequences of, differential use of this concept, and whether this connects to differences in the presence of the phenomenon or what, following Lagendijk et al. (2014: 358), might be described as ‘geographies of the articulation of the concept’ and ‘geographies of the phenomenon’ of rural gentrification. They suggest that assemblage theorizations foster comparative studies exploring ‘variations and complexities’ associated with use of the term gentrification. Such an approach has parallels with Latour’s (1999) concept of ‘circulatory sociologies of translation’ employed in Phillips’ (2007; 2010) explorations of the use of concepts of gentrification, class and counterurbanization within rural studies. Latour’s concept provides an effective way of developing comparisons that recognize the limitations and potentials of travelling theories.

**Figure 3.**
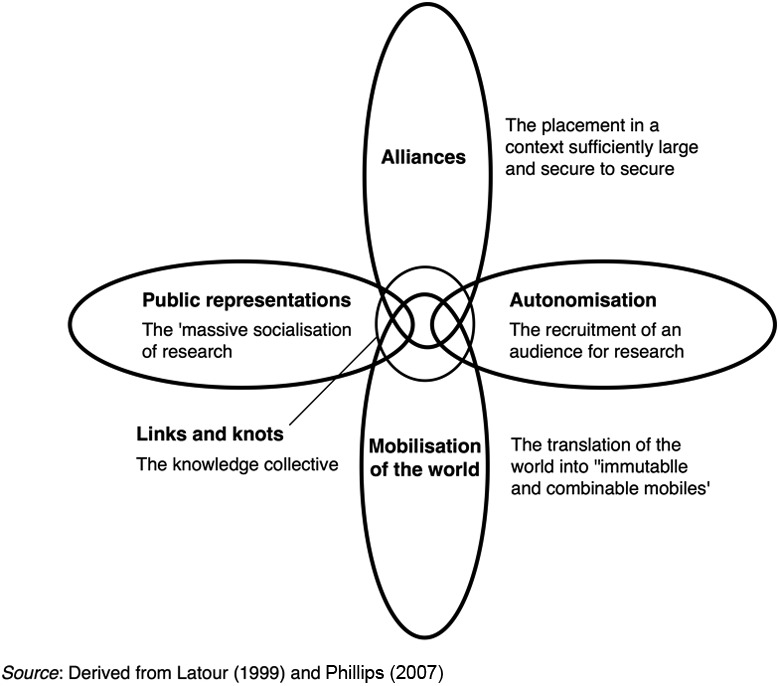
Circulating sociologies of translation.

Latour develops his concept of circulatory sociologies of translation as a way of ‘enumerating’ types of activities and actants that need to be enrolled in constructing concepts and knowledge. He argues that concepts are analogous to a ‘heart beating in a rich system of blood vessels’ (Latour, 1999: 108), being simultaneously at the centre of a circulating system and dependent on flows from other elements of the system. Drawing on this analogy, Latour argues that concepts be conceived as ‘links and knots’ at the centre of ‘loops’ of flow, or ‘circulating sociologies of translation’, which bring assets to sustain the development of the concept. These circulating sociologies are identified as autonomization, alliance building, public representation and mobilization (Figure 3).

### Autonomization

Latour (1999) describes the enrolment of support for a concept or interpretation within worlds of academic activity and discourse as autonomization. Although there are no detailed sociologies of rural studies (although see Murdoch and Pratt, 1993), studies pointing to the significance of autonomization in understanding differential levels of engagement with the concept of rural gentrification in France, United Kingdom and United States can be identified.

Kurtz and Craig (2009) and Woods (2009), for example, identify differential developments in UK and US rural geography. For Kurtz and Craig, the publication industry fostered differential engagements with theory, with UK rural studies being more theoretically inclined due to a focus on journal article and edited book production, while US rural studies were more empirically focused through an emphasis on regional book monographs. Woods (2009), while accepting this differentiation of rural geography, argued that processes of disciplinary institutionalization played an important role, creating in the United States a stronger theoretical orientation among rural sociologists than rural geographers, while UK rural geography became highly engaged in social theoretical debates in part because of institutional marginalization of rural sociology in this country. Thomas et al. (2011) provide a different account of the institutionalization of US rural sociology, stressing its severance from wider sociology. It is evident that geographers have more readily adopted the concept of rural gentrification than sociologists (although see Brown-Saracino, 2009; Hillyard, 2015), while its adoption within UK rural geography may reflect the significance of ‘political-economy’ perspectives in geography during the 1980s and 1990s. The subsequent turn towards culture that invigorated UK rural studies in the later 1990s also inspired considerations of the role of rural space as a motivator of rural gentrification (e.g. Phillips, 2002; 2014; Phillips et al., 2008; Smith and Phillips, 2001). Important disciplinary differences have been identified within French rural studies (Lowe and Bodiguel, 1990), although in both geography and rural sociology during the 1980s and 1990s, there was an emphasis on empirical studies, with limited engagement with social theory and epistemological reflections (Alphandéry and Billaud, 2009; Papy et al., 2012). This was despite notable French social theorists who have influenced gentrification studies in the Anglophonic world, such as Bourdieu and Lefebvre, undertaking early work in rural sociology (Elden and Morton, 2016; Phillips, 2015).

While differences in levels of theoretical reflection within disciplines at particular moments in time can influence engagement with conceptions of gentrification, other processes are also influential, including enrolment of other concepts. Fijalkow and Préteceille (2006) and Préteceille (2007), for example, argue that gentrification’s low uptake in France reflects a preference to use the concept of ‘embourgeoisement’, conjoined with concerns about the coherence and relevance of the gentrification concept within French contexts (cf. Rousseau, 2009). This preference may, however, have limited applicability within a rural context, where long-standing preoccupations with processes of agricultural change and the status of French peasants and small producers fostered disconnection with notions of embourgeoisement circulating in other social science discourses (Hervieu and Purseigle, 2008; Rogers, 1995).

Another influence on French rural studies was its framing of rural space as a passive subject of urban change. The countryside was viewed as losing its specificity (Berger et al., 2005), either becoming urbanized (sometimes described as rurbanization) or more differentiated, such that there were no clear lines of distinction between the urban and rural (Hervieu and Hervieu-Léger, 1979; Jean and Perigord, 2009; Kayser, 1990). Large areas are ascribed an urbanized identity, without consideration of landscape character or public perceptions (Mathieu, 1990; 1998). These ‘peri-urban’ areas include accessible localities akin to those that formed the locus of UK studies of rural gentrification (Figure 1). Similarly, in the United States, conceptions of the exurban and the rural as simply non-metropolitan may contribute to rural gentrification being applied primarily in areas with low levels of urban commuting (Figure 2), although as mentioned previously, consideration might also be given to the differences in the scale of areas being characterized as rural: according to the OECD’s (2016) ‘national area classification’, for instance, only 24.1% of the United Kingdom is designated as rural, compared to 77.8% and 40.9% of United States and France, respectively.

Simultaneous with academic movements towards recognition of the peri-urban in France was growing public interest in issues of rural cultural identity (Bonerandi and Deslondes, 2008). Paralleling these changes was movement from quantitative assessments of population numbers/movements to qualitative consideration of how these connect to transformations in popular understandings of the countryside. These include studies of international migrants in French rural places (Benson, 2011; Barou and Prado, 1995; Buller and Hoggart, 1994; Diry, 2008), as well as a few studies explicitly referencing notions of rural gentrification (Cognard, 2006; Perrenoud, 2008; Puissant, 2002). However, across all three countries, discussions have generally been framed in registers other than gentrification, with terms such as amenity migration, counterurbanization, neo-ruralism, peri-urbanization, rural renaissance and social segregation and differentiation being preferred over gentrification.

### Alliance building and public representations

Phillips (2010) has discussed relationships between conceptions of rural gentrification and counterurbanization, arguing that in UK and US studies, the latter gained strength over the former not only through widespread circulation within academic channels of autonomization but also through the circulatory sociologies of alliance building and public representation. Counterurbanization, it is claimed, drew strength from alignments with the intellectual contours of governmental statistics production and policymaking, while also making use of ‘social abstractions well embedded in, or highly commensurable with, public normative consciousness’ (Phillips, 2010: 553). Consequently, counterurbanization circulated relatively easily within public discourses, with Halfacree (2001: 400) highlighting how it ‘spun out into popular debate’, particularly within the United Kingdom, where narratives of residential migration to the countryside are reproduced across television documentaries and dramas, newspapers and popular fiction. The concept of gentrification, on the other hand, has social connotations of class that may have limited its uptake in public and policy contexts, although at times feeding into both (Phillips, 2002; 2004).

Applying such arguments to comparisons between the United Kingdom, France and United States suggests that circulatory sociologies of alliance building and public consciousness, as well as autonomization, may significantly differ. Reference has, for example, already been made to the significance of concepts such as peri-urbanism within French rural studies, and this concept gained significant academic impetus when included as a category in the *Institut National de la Statistique et des Études Économiqu*e official classification of French national spaces in 1996 (Le Jeannic, 1996). This change both reflected the conceptual success of the peri-urban within academic debates and institutionalized the peri-urban as a category of space deserving not only academic attention but also as a subject for political and public discourse, although with respect to the latter, notions of urban and rural space still predominate. Similar arguments can be made with respect to the *US General Accounting Office* that classifies land using categories (e.g. urban, urbanized, urban cluster, metropolitan, micropolitan, nonmetropolitan and rural) that effectively cast the rural and nonmetropolitan as residual classifications with no consideration given to their material character or public perceptions of these areas. In the United Kingdom, by contrast, governmental spatial classifications have, at least in England and Wales, demonstrated parallels to aspects of popular constructions of rurality since 2004 (cf. Bibby and Shepherd, 2004; Bibby and Brindley, 2016; Phillips et al., 2001). One consequence is that areas close to urban areas have been identified as locations of ‘rural’ gentrification (Figure 1).

There is evidence pointing to greater popular and policy engagement with the term gentrification in North America than in the United Kingdom or France. Guimond and Simiard (2010), for example, suggest that rural gentrification attracted the attention of television producers, as well as reporters, in Quebec’s provincial and regional press. In the United States, rural gentrification research by Nelson figured in an article in the *Wall Street Journal* (Dougherty, 2008), while in relation to alliance building, the *Housing Assistance Council*, in co-operation with *US Department of Housing and Urban Development*, produced a high-profile report on rural gentrification (Housing Assistance Council, 2005). Furthermore, while the term rural might not be applied by academics and policymakers to areas with high commuting to large urban areas, there are numerous cases of literary and filmic representations of such spaces that enact motifs of rurality and gentrification.

Part of the policy interest in rural gentrification within the United States links to what has been described in urban studies as ‘positive gentrification’ (Cameron, 2003), whereby state agencies perceive there to be benefits from processes of gentrification, such as the influx of capital-rich migrants whose consumption, skills and enterprise might stimulate local development and employment. While subject to considerable criticism within urban studies (Smith, 2002b; Slater, 2006), this conception of rural gentrification has resonances with studies of migration to non-metropolitan areas in the American West (Beyers and Nelson, 2000; Gosnell and Abrams, 2011; Nelson, 1999), to Stockdale’s (2006; 2010) work on rural gentrification and the impacts of rural in-migration in Scotland, and to the activities of some French local authorities which have sought to attract particular in-migrants, such as entrepreneurs or other ‘project backers’ (Richard et al., 2014).

In relation to public representations, Lamont (1992; 2000) and Bennett et al. (2009) suggest there is greater acceptance of notions of hierarchical differentiations in cultural value in France than in the United Kingdom or United States, and conversely, less receptivity to identities constructed around socio-economic distinctions. Such arguments are of clear importance to the study of gentrification given that research has suggested that symbolic distinctions are of crucial significance to its formation (e.g. Butler and Robson, 2003; Rofe, 2003). Furthermore, connections between cultural values and academic interpretations of society have been highlighted by Savage (2010), who presents an historical account of changing concepts of culture within the UK middle classes, connecting these to developments in the conduct of sociology. Among the studies used to develop this argument was Pahl’s (1965) research on Hertfordshire villages, which has been viewed as constituting a study of rural gentrification by people such as Paris (2008), despite it making no use of the term. For Savage, Pahl’s study represents both a description and enactment of technocratic middle-class culture (Phillips and Smith forthcoming). Circulatory sociologies of translation are, however, often far from direct: Although the concept of gentrification appears not to have translated readily into French public and academic discourse, the writings of French social theorists such as Bourdieu, Latour, Lefebvre and Waquant have exerted a profound influence on UK and US gentrification studies (e.g. Bridge, 2006; Butler and Robson, 2003; Phillips, 2010; 2015), although not on French rural studies.

### Mobilization

The final circulating sociology identified by Latour (1999: 108) relates to practices and processes of inscription and translation through which objects of study become ‘progressively loaded into discourse’. This circulation has long been the focus of epistemological and methodological discussion about the ability, or not, of concepts to connect to objects or situations, issues that have been, and continue to be, a focus of debate within gentrification studies. While there have been claims that the ontological debates over the meaning of the concept of gentrification have declined in significance (e.g. Lees et al., 2008; Slater et al., 2004), the rise of comparative research has certainly challenged this, with Ghertner (2015: 552), for example, arguing that the concept ‘fails in “much of the world”’. This argument, advanced in relation to studies of the Global South, has relevance even within the studies of the metropolitan North, given that there are both variegated understandings of the concept and numerous criticisms raised about its value. The complex geography to the adoption of the concept has been neglected both by its exponents and critics, as evidenced by use of the term rural gentrification, which not only is far from extensive in France but is also relatively limited even in the United Kingdom and United States.

While processes of autonomization, alliance building and representation may profoundly influence the acceptance and development of the concept of rural gentrification, differential recognition of the concept in France, United Kingdom and United States may also reflect differences in the activities and dynamics of change occurring in the countryside in these countries. As such there is a need to conduct comparative research exploring if conceptions of rural gentrification provide differentially effective mobilizations of the rural ‘pluriverse’ (Latour, 2004: 40) in each country, or as it might also be expressed, to explore the geographies of the phenomenon, or phenomena, of rural gentrification, as well as its articulations. Clearly, given earlier discussions, there are a host of practical, methodological, epistemological and political issues to be considered in developing such comparative research. In the context of the present article, however, we will restrict ourselves to considering how Tilly’s (1984) typology of strategies of comparison, along with Robinson’s (2015) differentiation of genetic and generative comparisons, could assist in mobilizations of conceptions of gentrification applicable across rural France, United Kingdom and United States, as well as being of potential wider relevance in studies of gentrification.

### Genesis and generation within strategies of comparisons

It has been argued that many studies of rural gentrification implicitly adopt an individualizing comparative perspective, although evidence of national differentials in the focus of studies (Figures 1 and 2) indicates potential for variation-finding comparisons exploring whether differences reflect the influence of contextual processes such as landscapes, planning regulations or property relations. Darling’s (2005) work was discussed in relationship to the former, while UK studies have identified the latter two as important influences on the geography of rural gentrification, particularly its focus within smaller rural settlements (Phillips, 2005). Studies in the United States also highlight the significance of rural gentrification in transforming property and land-management practices (Abrams et al., 2013; Gosnell and Travis, 2005).

Such work does not preclude identification of contextually specific understandings and practices and, when combined with analysis of the sociologies of translation operating within such contexts, can produce insights that speak back to prevailing conceptualizations of gentrification. Robinson (2015) argues for the development of comparative approaches that combine ‘genetic’ and ‘generative’ tactics of conceptual development. The former, as previously discussed, examine the genesis or emergence of seemingly common/repeated or related outcomes, while the latter explore how examination of ‘different singularities or cases’ generate insights and problems that provoke new lines of thought that can potentially be bought ‘into conversation’ with prevailing conceptualizations. These conversations might, as in individualizing comparisons, centre around differences between cases, although Robinson sees scope for generating connections which resonate across and from cases and hence can be of value within other strategies of comparison.

Gentrification studies provide illustrations of such conversations. Focusing on the application of stage interpretations, a past conversation will be outlined, before considering a hitherto rather implicit one and one in need of development. In relation to the first, although, as previously argued, stage models are commensurable with universalizing and encompassing comparisons, they have been created generatively. Early-stage models of urban gentrification emerged from comparisons between inner-city locations in North America (e.g. Clay, 1979; Gale, 1979). Later-stage models (e.g. Hackworth and Smith, 2001; Hackworth, 2007; Lees et al., 2008) drew on different theoretical understandings of gentrification and from recognizing forms of gentrification that differed from the ‘classical’ gentrification of the 1960s to 1980s, which came to be viewed as a ‘pioneer’ phase of gentrification, involving small-scale sporadic transformations of buildings. Pioneer/classical/sporadic gentrification became, and very much still act, as comparators to set against other forms of gentrification.

A second generative conversation that gentrification studies should recognize is that stage interpretations are more multidimensional than often represented. Work of people such as Rose (1984) on ‘marginal gentrification’, for example, promoted differentiation of gentrification on the basis of assets or capital. Marginal gentrifiers, often associated with the onset of gentrification, were viewed as having limited amounts of economic capital yet relatively high levels of cultural capital. They were seen to be frequently displaced by an ‘intensified gentrification’, involving larger scale, more professional and capitalized agencies, and gentrifiers with more economic capital and, at least relatively, less cultural capital. In some locations, gentrification was seen to extend in scale to encompass not only large areas of residential properties but also other transformations, with Smith (2002b: 443) coining the phrase ‘gentrification generalised’ to refer to the formation of ‘new landscape complexes’ whereby not only housing but also ‘shopping, restaurants, cultural facilities,…open space, employment opportunities’ become gentrified. This form of gentrification was widely associated with the construction of new-build properties and heightened involvement of state agencies, but has also been connected, within the work of Ley (1996), Butler and Robson (2003) and Bridge (2001; 2003), with a further decline in the significance of cultural capital as a ‘channel of entry’ (Phillips, 1998) into gentrified spaces. Some areas have also been identified as undergoing ‘super-gentrification’ (Butler and Lees, 2006) involving people with very high levels of economic capital.

Concepts such as economic and cultural capital facilitate universalizing comparisons through simplifying or ‘abbreviating’ (Robinson, 2015) the complexity of everyday life by focusing on particular, repeated aspects. Given this, it is unsurprising that studies of the UK countryside have made comparisons between stages and assets identified in urban studies and processes of change observed in rural areas (Phillips, 2005; Smith, 2002a). It appears that many UK rural localities have experienced intensified and generalized gentrification, given their high levels of middle class residence (Phillips, 2007). In the United States, the ‘American West’ has been a focus of attention within rural gentrification studies (Figure 3), and according to Nelson and Nelson (2010), is an area where it appears most widely present, although also occurring more sporadically across rural areas in the Mid-West, the South and the Eastern seaboard. Even in the American West, however, rural gentrification is shown to be concentrated in a relatively small number of areas, with Hines (2012: 75) likening its geography to an ‘archipelago’ of change set within ‘the midst of a relatively static, conservative, agricultural/ industrial “sea”’. In France, the progress of rural gentrification appears even more sporadic, as well as widely perceived via other process descriptors, such as international or neo-rural in-migration, tourism or peri-urban or new-build development. A study of the High Corbières has, however, suggested that neo-rural migration reflected an early sporadic phase of gentrification which was followed by inflows of people with both more economic assets and greater levels of cultural capital (Perrenoud and Phillips, forthcoming).

**Figure 4.**
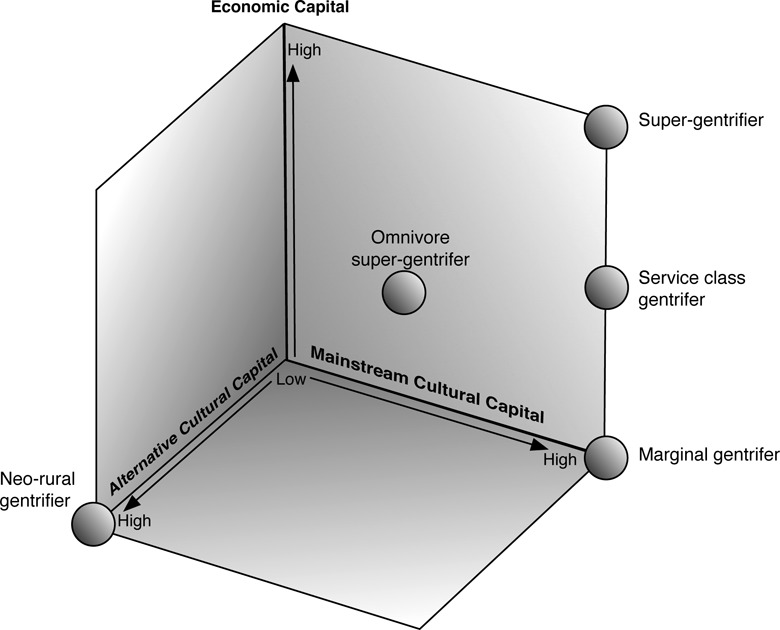
Gentrifiers within a 3-dimensional asset-based theorisation.

Such research highlights that comparisons can generate connections between studies of rural gentrification and investigations framed through other concepts. They also point to how more multidimensional understanding of gentrification could be constituted by recognizing that economic and cultural capitals take a range of different forms. Ley (1996), for example, argues that gentrification can be associated with ‘critical’ or ‘counter-cultural values’. As outlined in Phillips (2004), such arguments have rural counterparts, not least in the work of Smith and Phillips (2001) which highlighted the presence of what they characterize as ‘New Age professionals’. Smith subsequently developed this argument further, highlighting how some areas are experiencing gentrification sparked and reproduced by householders seeking to realize a range of ‘alternative’ ways of living (Smith, 2007; Smith and Holt, 2005). These arguments chime with aspects of Hines’ (2010; 2012) work in a North American context, as well as notions of neo-rural migration employed in France. Drawing on such arguments, it can be argued that some capital/asset-based analyses of gentrification employ what could be described as a three-dimensional differentiation of gentrifiers and gentrification (Figure 4).

Three-dimensions, however, are insufficient, an argument that can be illustrated by considering the concept of ‘super-gentrification’. This concept, which has been briefly discussed in a rural context by Stockdale (2010) and potentially has wider relevance, both within rural areas close to global cities such as London, Paris and New York and to remote amenity locations, has generally been used to describe people who are ‘super-rich’ in economic terms. However, studies suggest that there are a range of cultural dimensions that need fuller investigation. Super-gentrification, for example, has been identified with practices of conspicuous consumption, with Lees (2003: 2487) arguing that it involves ‘intense investment and conspicuous consumption by a new generation of super-rich “financifiers”’. As such super-gentrification can be seen to connect to objectified forms of cultural capital (Bennett et al., 2009; Bourdieu, 1986), which, as Phillips (2011) has observed, can be used to frame much of the analysis of culture and class conducted within UK rural studies in the 1980s and 1990s.

Butler and Lees (2006), however, also suggest that, at least in the Barnsbury area of London, super-gentrifiers were predominately drawn from elite segments of the British education system (i.e. public or selective secondary schools and Oxbridge). As such, these gentrifiers had high levels of credentialed or institutional capital (Bourdieu, 1986) but also enact a range of embodied forms of cultural and social capital reproduced through this educational system (Bennett et al., 2009; Savage, 2015). Such connections are not universal, with Butler and Lees (2006) drawing contrasts between their study and the work of Rofe (2003) and Atkinson and Bridge (2005) on the habitus of gentrifiers in other global cities, which appear to be more cosmopolitan in origin and cultural orientation. Savage, in a series of works (Bennett et al., 2009; Savage et al., 1992, 2013), has argued for recognition of a range of different forms of cultural evaluation beyond the classical high–low distinction (see also Lamont, 1992; 2000; Warde and Gayo-Cal, 2009). In some contrast, Perrenoud and Phillips (forthcoming) argue that rural areas of southern France are experiencing gentrification by people connected to the production of Parisian ‘high culture’, and who might be described as ‘super-gentrifiers’ in a cultural sense, as well as being well endowed with economic assets. Even within the study of super-gentrification, there is a need to move analysis beyond three-dimensions, to recognize a range of different forms of cultural capital, an argument advanced more generally in relation to studies of rural gentrification by Phillips (2015). Also, earlier work (Phillips, 2004) on a ‘composite’ stage-interpretation of rural gentrification highlighting labour, property and finance capital flows, provides an example as to how multidimensionality can be applied to the concept of economic as well as cultural capital.

Comparison holds the potential for fostering the creation of more multidimensional asset-based studies of gentrification. Petersen’s discussions of cultural omnivores provide an interesting example of this, not only suggesting that the concept of people engaging in both high and mass cultural activities could link into gentrification (Petersen and Kern, 1996), but also highlighting its emergence from comparative work inspired by Bourdieu’s writings and how it catalysed critiques and revisions of Bourdieu’s conceptualizations of cultural capital (Petersen, 2005).

Concepts of capital and flow point to relationality, which is a third generative conversation that gentrification studies should develop. As outlined earlier, relationality is central to encompassing comparisons. However, as Wright (2015) has observed, there is considerable variability of relationality evident within so-called relational perspectives. He, for example, argues that the capital-based theorization of class developed by Savage et al. (2013) is an example of an ‘individual-attributes’ based approach that pays insufficient attention to the way that holding and use of assets by one person can causally connect to those of other people. He identifies more relational perspectives focused around the hoarding/closure of opportunities and relations of domination/exploitation, but his analysis is explicitly centred on economic conditions and activities. Consequently, he does not provide a template for developing multidimensional asset-based studies of gentrification, but his discussion of forms of relationality are significant, not least because they highlight the need to situate analysis of assets held by individual agents of gentrification into examinations of their relationships within wider fields. The designation of levels of capital held or required for gentrification, for instance, clearly varies according to the context in which they are being deployed. Rural studies, for example, have routinely made reference to migration as an opportunity to maximize the purchasing power of financial assets held by householders, be this through voluntary or induced down-sizing or through up-sizing via purchasing housing in areas where prices are lower than at current place of residence (Smith and Holt, 2005; Stockdale, 2014). There are also less widespread references to the significance of the spatial transferability or fixity of cultural qualifications and competencies (Cloke et al., 1998a; Fielding, 1982). Connections could be forged between this work and wider discussions of migration and cultural capital, particularly those, such as Erel (2010), that highlight the need to consider not only amounts and forms of capital migrants move with, but also how these are reconfigured and created through interactions in new locations of settlement.

Overall, there appears to be considerable value in recognizing the genetic and generative role of comparisons across all the strategies of comparison identified by Tilly and indeed to employ all these strategies when seeking to mobilize conceptions of gentrification in relation to rural France, United Kingdom and United States. Among the implications of this perspective is that there are variations in both the strategies of Tilly and tactics of Robinson, and careful consideration needs to be paid to how these fold into each other as comparative studies are developed.

## Conclusion

Taking debates within urban studies about gentrification and comparison as a starting point, this article has investigated how comparative studies of rural gentrification can be advanced. Drawing attention to Tilly’s (1984) identification of individualizing, universalizing, encompassing and variation-finding strategies of comparison, the article identified elements of each in studies of rural and urban gentrification, before exploring how they can be developed within a comparative study of rural gentrification in France, United Kingdom and United States.

The article has compared the uptake of the concept of rural gentrification through Latour’s (1999) concept of circulatory sociologies of translation. Attention was drawn to the emphasis on theory, and particularly political-economic theories, within UK rural studies as compared to France and United States during the late 1980s and 1990s, facilitating engagement with concepts such as class and gentrification. UK geography also underwent a ‘cultural turn’ that encouraged explorations of rural space as a motivator of in-migration and of contestations between different residential groups. Such concerns were not just relevant to conceptualizations of rural gentrification, and across all three countries, other concepts more successfully enrolled advocates. In part, this success stemmed from alignment with the demands of other circulatory sociologies connected to governmental statistics production, policymaking and popular discourses. Cross-national differences may be significant, with rural gentrification obtaining greater popular and policy engagement in United States than in France or United Kingdom.

Such differences play key roles in concept development and application, and the extent to which gentrification articulates with or mobilizes the world. Described by Latour as the circulatory sociology of mobilization, this aspect of concept development can be framed in terms of relationships between geographies of the concept of rural gentrification and geographies of the phenomenon of gentrification. More specifically, differences in the recognition of rural gentrification in France, United Kingdom and United States might reflect differences in extent and form of gentrification occurring in these countries, as well as differences in the circulatory sociologies of autonomization, alliance-building and public representations.

Addressing such issues requires consideration of strategies of comparison. While adoption of a variation-finding strategy is difficult due to the small number of rural gentrification studies, and indications of a preference for individualizing comparisons among recent rural studies are evident, it is possible to identify arguments for adopting variation-finding, relational and universalizing strategies of comparison in rural gentrification research. In relation to variation-finding comparisons, the value of comparing gentrification across different types of rural areas was noted, an argument that could be extended to encompass comparisons across urban and rural spaces. The benefits of investigating national differences in planning regulations and property relations, and their role in conditioning the geographies of rural gentrification, were also highlighted.

Variation-finding comparisons involve acceptance of elements of commonality across the cases being investigated, both with respect to the identification of generic contours of processes and the formation of contextual variation. There are connections here to universalizing perspectives. While universalizing approaches have been criticized as decontextualist, reductionist and developmentalist, viewing then ‘genetically’, as repetitions whose emergence always needs to be explained, avoids establishing a universalizing approach that creates ‘concepts without difference’ or an individualizing approach that establishes ‘difference without conceptualization’ (Robinson, 2015: 17).

Employing a genetic approach can not only reinvigorate universalizing comparisons but can also be incorporated into individualizing, variation-finding and relational or encompassing comparisons as well. Furthermore, Robinson’s highlighting of the generative role of comparisons within studies of gentrification is valuable. Focusing on stage interpretations of gentrification, three examples of generative comparisons were discussed, linked to their significance in their emergence, their role in fostering multidimensional understandings of gentrification and the potential value of recognizing different forms of relationality. Such examples reveal that rather than adopting a singular strategy or tactic of comparison, there is a value in employing them in combination.

This article is the first to reflect on the merits of comparative approaches to the study of rural gentrification. Although focused on the development of a cross-national study of rural gentrification, we have framed our explorations through comparative engagements not only with studies of rural space but also with ideas from urban and wider geographical studies. This framing reflects, in part, two aspects of comparison highlighted by McFarlane (2010: 725). First, it enacts ‘comparison as learning’, as we have drawn upon literatures addressing issues that are, as yet, largely omitted from the discourses of rural studies. Second, it also involves an ethico-political impetus for comparison, in that we hope that our discussion would indeed ‘speak back’ to centres from where we have drawn insight, not least in raising questions about the metrocentricity of contemporary discussions of comparative research.
